# Is there still need for a further randomised controlled trial on the route of hysterectomy for benign disease?

**DOI:** 10.52054/FVVO.13.2.024

**Published:** 2021-06-28

**Authors:** Clark T.J., Saridogan E

**Affiliations:** Birmingham Women’s Hospital; University College London Hospital

One in ten women undergo a hysterectomy during their lifetime, mostly for benign conditions (Nieboer et al., 2009). Prior to the introduction of laparoscopic hysterectomy by Harry Reich in 1989 (Reich 1992), vaginal and abdominal routes were the two main methods for the removal of the uterus. In the last three decades the proportion of hysterectomies performed by the laparoscopic route has gradually increased. In England during 2015-6, three abdominal hysterectomies were done for every laparoscopic hysterectomy for benign indications excluding genital prolapse (Royal College of Obstetricians and Gynaecologists, 2018). A more recent assessment that analysed data from all hospitals in England between 2011-2017, and included both benign and malignant indications, showed that nearly 50% of hysterectomies are now performed by laparoscopy (Madhvani et al., 2019). The rates of laparoscopic hysterectomy increased despite national guidance from the National Institute for Health and Care Excellence (NICE) recommending vaginal and abdominal hysterectomies over the laparoscopic route for safety reasons (National Institue for Health and Care Excellence, 2007). This recommendation was based upon systematic reviews showing a higher rate of severe bleeding and urological complications (Aarts et al., 2015).

The latest Cochrane review from 2015 (Aarts et al., 2015) identified 25 trials, including a total of 2983 women, comparing laparoscopic and abdominal hysterectomy for benign indications. Laparoscopic hysterectomy was found to have significantly more urinary tract injuries (bladder or ureter) but the available evidence was of low quality. Moreover. the largest RCT included in this review was conducted over 15 years ago, when laparoscopic hysterectomy was in its infancy (Garry et al., 2004). The latter trial was criticised for operator bias, with differential expertise between established abdominal techniques and newer laparoscopic approaches (Donnez et al., 2004, Chien et al., 2005).

Despite the outstanding uncertainties, many gynaecological endoscopic surgeons believe that there is no debate about the preferential route of hysterectomy for most benign conditions (personal communication Clark TJ), instead research into laparoscopic hysterectomy should focus on improving outcomes through technical adjustments and other interventions to enhance recovery. One such study is published in this issue of Facts, Views and Vision by Marwah et al (2021). They present a retrospective comparison of two different methods of haemostasis during laparoscopic hysterectomy in almost 600 women; intracorporeal endosuturing and energy based coagulation. They found that the post-operative outcomes including operative time, blood loss, pain score after surgery, mean post-operative stay and complications were comparable between the two groups.

Since the conduct of the largest RCT comparing laparoscopic, open and vaginal hysterectomy (Garry et al., 2004), there have undoubtedly been significant improvements in the energy devices that are used for vessel sealing, which include advanced bipolar and ultrasound energy. Energy devices are the commonest method for sealing vessels in current clinical practice although the wider availability of laparoscopic suturing courses combined with an increased laparoscopic caseload in general, is increasing the number of surgeons able to use laparoscopic sutures. The safety and recovery findings by Marwah et al (2021) in this issue of FVVO demonstrates the equivalence of both techniques in surgeons with the appropriate expertise. Marwah et al discuss the potential for non-target thermal injury and devascularisation of tissues associated with energy devices. However, modern energy devices minimise the likelihood of thermal spread. In addition, barbed, self-locking sutures to close the vaginal vault have made laparoscopic hysterectomy more attainable for surgeons without enhanced skills in intracorporeal suturing. Thus, the findings from Marwah et al suggest that there is no preferential technique for those with the requisite suturing skills. However, gynaecologsists without such skills can be reassured that the adoption of advanced energy modalities will provide equitable outcomes. Thus, the need to acquire intracorporeal suturing skills is not essential for best outcomes in laparoscopic hysterectomy but surgeons should be encouraged to acquire such skills to broaden their laparoscopic surgical repertoire and also to deal with complications such as inadvertent visceral injury.

Returning to the broader debate about route of hysterectomy for benign conditions as opposed to evaluation of technical modifications alone, we need to understand why national and international guidelines still advising caution against laparoscopic hysterectomy? The simple answer is probably the fact that as gynaecological endoscopic surgeons we have not provided good enough clinical evidence to reverse the legacy of earlier studies suggesting increased complications with the laparoscopic approach. Previous findings that laparoscopic hysterectomy takes longer and has a higher complication rate may no longer be valid within the context of contemporary gynaecological practice. This is because training in addition to advances in surgical equipment has vastly improved since these studies were done and familiarity with laparoscopic hysterectomy has increased. Hence, we believe that there is a need to conduct a new RCT to determine the best route for hysterectomy in 2021 for benign indications. Survey data from both clinicians and patients rank the major complication rates as the most important deciding factor in determining the route of hysterectomy (personal communication Clark TJ). Hence, such studies will need to be powered to demonstrate non-inferiority of laparoscopic hysterectomy in terms of complications.

In the United Kingdom the National Institute of Health Research (NIHR) has approved funding of such a trial. The LAVA (LAparoscopic Versus Abdominal hysterectomy) trial will compare laparoscopic with conventional abdominal hysterectomy (https://fundingawards.nihr.ac.uk/award/NIHR128991). Vaginal hysterectomy has been shown to be beneficial in terms of complications and recovery but this technique is largely confined to women with prolapse and where the uterus is not enlarged. This leaves a choice between laparoscopic and conventional abdominal hysterectomy for the majority of hysterectomies for benign indications and it is this specific comparison that the LAVA trial will evaluate.

The LAVA trial has specific strengths to inform clinical practice. Firstly its size and generalizability; the trial will aim to randomise 3250 women from at least 50 centres and will be the largest ever trial of its kind on this subject, exceeding the total number of patients randomised from 25 RCTs in the latest Cochrane review on the subject (Aarts et al., 2015). Secondly, the LAVA trial will adopt an expertise-based design (Cook et al., 2015). This means that recruiting centres will be required to ensure that the type of hysterectomy allocated after randomisation is only performed by pre-identified surgeons experienced in that particular technique. This will help overcome the biases prevalent in some previous trials where increased complications rates of laparoscopic hysterectomy may have been attributable to participating surgeons being relatively more experienced in abdominal or vaginal hysterectomy. Thirdly, the LAVA trial is pragmatically designed. Women are eligible if they have a benign gynaecological condition(s) requiring a hysterectomy and are suitable for either surgical technique; this “suitability” is decided by the surgical team within each participating centre. Finally, the impact of reduced trauma from avoiding laparotomy on recovery may have been underestimated. This is because short and medium term recovery after hysterectomy has invariably been assessed using blunt, ill-defined and insensitive measures such as “return to work”, “resumption of normal activities” and generic quality of life tools. Full recovery will be a key secondary outcome of the LAVA trial, which will be measured using a novel, personalised recovery tool completed by text over the weeks following surgery (Rose et al., 2014; van der Meij et al., 2018).

Whilst globally, the uptake of laparoscopic hysterectomy has been slow, the situation is changing with greater familiarity, better training, better equipment and increased proficiency in the technique, such that nearly as many hysterectomies for benign disease are now being done laparoscopically as abdominally (Madhvani et al., 2019). These changes, and the ongoing uncertainty about the advantages and disadvantages of laparoscopic compared with abdominal hysterectomy, particularly the relative rate of complications of the two procedures necessitates a large, robust, multi-centre RCT to compare contemporary laparoscopic hysterectomy with abdominal hysterectomy to determine the safest and most cost-effective technique. The LAVA trial hopes to overcome the main methodological shortcomings of previous trials to provide a definite answer on the safety of laparoscopic hysterectomy compared to the open route and its impact upon post-operative recovery.
